# Places of Persistence: Slavery and the Geography of Intergenerational Mobility in the United States

**DOI:** 10.1007/s13524-018-0693-4

**Published:** 2018-07-03

**Authors:** Thor Berger

**Affiliations:** Department of Economic History & Centre for Economic Demography, Lund University, Scheelevägen 15B, 223 63 Lund, Sweden

**Keywords:** Intergenerational mobility, Slavery, Persistence

## Abstract

Intergenerational mobility has remained stable over recent decades in the United States but varies sharply across the country. In this article, I document that areas with more prevalent slavery by the outbreak of the Civil War exhibit substantially less upward mobility today. I find a negative link between prior slavery and contemporary mobility within states, when controlling for a wide range of historical and contemporary factors including income and inequality, focusing on the historical slave states, using a variety of mobility measures, and when exploiting geographical differences in the suitability for cultivating cotton as an instrument for the prevalence of slavery. As a first step to disentangle the underlying channels of persistence, I examine whether any of the five broad factors highlighted by Chetty et al. (2014a) as the most important correlates of upward mobility—family structure, income inequality, school quality, segregation, and social capital—can account for the link between earlier slavery and current mobility. More fragile family structures in areas where slavery was more prevalent, as reflected in lower marriage rates and a larger share of children living in single-parent households, is seemingly the most relevant to understand why it still shapes the geography of opportunity in the United States.

## Introduction

Intergenerational economic mobility is lower in the United States than in most other developed countries and has remained fairly constant over recent decades (e.g., Chetty et al. 2014b; Corak 2013; Lee and Solon 2009; Mazumder 2005; Solon 1992).^1^ However, large differences in mobility exist within the country. For example, some areas in the Midwest exhibit rates similar to those observed in high-mobility Scandinavia, but places in the Southeast are among the least mobile in the developed world (Chetty et al. 2014a). A sharp regional divide in the extent to which economic status is transmitted between generations and the relatively stable spatial variation in upward mobility over time point to local historical factors as potentially important for understanding the geography of opportunity in the United States.

In this article, I examine whether differences in intergenerational mobility across the United States reflect the historical distribution of slavery. Against the backdrop of a growing empirical literature documenting how past events shape present-day outcomes surveyed in Nunn (2009), I ask whether the legacy of slavery has detrimental long-run effects on upward mobility. By pairing county-level data from the 1860 census containing information on the prevalence of slavery with recent mobility estimates for commuting zones (CZs) from Chetty et al. (2014a), I show that CZs where slavery was more prevalent by the outbreak of the Civil War exhibit considerably less upward mobility today.^2^

**Fig. 1:**
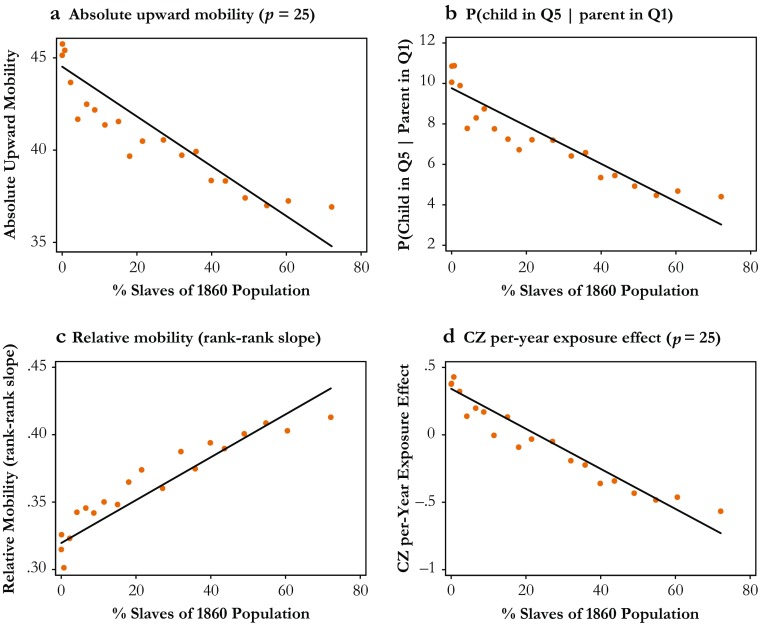
Slavery and mobility in the United States. These figures show binned scatterplots of the CZ–level relationship between the percentage of the population enumerated as slaves in the 1860 census and four alternative mobility measures for children born in the early 1980s: (1) the expected income percentile at age 30 for children born to parents at the 25th percentile of the national income distribution (panel a); (2) the probability that a child born to parents in the bottom income quintile ends up in the top quintile in adulthood (panel b); (3) the rank-rank slope of child and parent income ranks (panel c); and (4) the causal place effect of each CZ on adult income for children born to parents at the 25th income percentile (panel d). To construct each figure, all 499 CZs in the main sample are collapsed into 20 bins based on the share of the population enumerated as slaves, and for each bin the mean of each respective mobility measure is depicted. The first bin contains all CZs with no slaves recorded in the 1860 census, and each subsequent bin contains approximately 15 CZs. Also shown are fitted OLS regressions based on the underlying (ungrouped) CZ–level data

Figure 1 illustrates the CZ–level link between the share of the population enumerated as slaves in the 1860 census and several complementary mobility measures (described in more detail in the next section). Consistently, these binned scatterplots suggest that intergenerational transmission of economic status is higher in areas with more prevalent slavery and that children growing up in these CZs have substantially lower expected earnings in adulthood relative to children born to parents at similar income levels elsewhere in the country. For example, the average probability that a child born to parents in the bottom income quintile will reach the top quintile in adulthood is roughly 4 % in the CZs with the highest prevalence of slavery, whereas it exceeds 10 % for children in CZs with no recorded slaves in 1860 (see Fig. 1, panel b).

Although the correlations in Fig. 1 are highly suggestive, the negative link between slavery and upward mobility may simply reflect a confounding factor, such as lower historical levels of industrial development or a more unequal distribution of income and wealth in the American South rather than slavery per se. However, conditioning on a wide range of historical and modern development outcomes leaves the negative link between slavery and upward mobility largely unaffected, suggesting that it does not mainly reflect the fact that places exposed to slavery were (or are), on average, less economically developed. A related concern is that the observed relationships may arise from a sorting of families with worse potential mobility outcomes into areas with more prevalent slavery, rather than reflect a causal effect of place. Using data from Chetty and Hendren (2016a, 2016b) to estimate the causal effect of each CZ on upward mobility net of sorting, however, I show that places with more prevalent slavery indeed exhibit negative place-level effects on the opportunity for economic advancement among children from low-income families, as depicted in Fig. 1, panel d. Last, to further support a causal interpretation of this relationship, I document that this link is also evident when using within-state variation in slavery and mobility, limiting the sample to the 15 historical slave states at the eve of the Civil War, and exploiting geographical differences in the suitability for growing cotton as the basis for an instrumental variables (IV) strategy.

As a starting point to understand why places with more prevalent slavery produce worse mobility outcomes, I conclude by examining the extent to which the five factors highlighted by Chetty et al. (2014a) as the most important correlates of upward mobility within the United States—family structure, income inequality, school quality, segregation, and social capital—weaken this link. More fragile family structures, as reflected in a lower share of married adults and a higher share of children living in households headed by single mothers, are seemingly the most important of the five candidates in accounting for the fact that upward mobility is lower in areas with a higher prevalence of slavery. Although this result should be interpreted carefully because of the challenges involved in identifying the precise underlying causal mechanisms and because multiple channels of transmission are likely at work, it is seemingly consistent with prior work emphasizing the adverse impact of slavery on the evolution of family structures in the American South after the Civil War and the historical continuity of family patterns (e.g., Gordon and McLanahan 1991; Miller forthcoming; Morgan et al. 1993; Ruggles 1994).

Together, these findings constitute new evidence that slavery has a causal negative impact on upward mobility, thus contributing to a theoretical and empirical literature arguing that slavery negatively affected subsequent local economic development and exacerbated inequality (Bertocchi and Dimico 2014; Lagerlöf 2005; Mitchener and McLean 2003; Nunn 2008b; Sokoloff and Engerman 2000), as well as persistently altered political attitudes among Southern whites (Acharya et al. 2016). Despite recent literature examining historical patterns of mobility (e.g., Collins and Wanamaker 2015; Long and Ferrie 2013; Olivetti and Paserman 2015; Sacerdote 2005), this is the first study to document a persistent impact of slavery on spatial differences in upward mobility, thus speaking directly to recent literature analyzing the geography of opportunity in the contemporary United States (e.g., Chetty et al. 2014a; Chetty and Hendren 2016a, 2016b) as well as to a broader literature documenting the long-term impacts of slavery and other exploitative historical labor-market institutions (e.g., Dell 2010; Nunn 2008a; Nunn and Wantchekon 2011).

## Measuring Mobility

A straightforward way to characterize the spatial variation in intergenerational mobility is to estimate income differences in adulthood for children born to parents in different locations but at a similar rank in the national income distribution. To obtain such measures, Chetty et al. (2014a) drew on federal income tax records for more than 10 million children born in the early 1980s, which they linked to their parents. Merging information on parental household income with children’s family income in adulthood (when they are approximately age 30) allows for a characterization of mobility patterns in nearly all CZs in the United States.^3^

In the main analysis, I use three complementary measures of mobility derived in Chetty et al. (2014a) that have been made publicly available at the CZ level through the Equality of Opportunity Project.^4^ First, I examine differences in absolute upward mobility across CZs captured by the mean rank at age 30 in the national (child) income distribution for children born to parents at the 25th percentile (*p* = 25) of the national (parent) income distribution.^5^ Second, a complementary measure of upward mobility is the probability that a child born to parents in the bottom quintile of the income distribution reaches the top quintile in adulthood, which is commonly interpreted as the chance to achieve the so-called American Dream. As shown in Table 1, panel A, a child born to parents at the 25th percentile, on average, reaches the 42nd percentile at age 30; a child born in the bottom quintile has, on average, 8.34 % chance of reaching the top quintile across the CZs in the main sample. Third, I examine spatial differences in relative mobility, which is captured by the slope in a regression of children’s income rank on parents’ income rank in each CZ, similar in spirit to conventional intergenerational elasticity (IGE) of income estimates for which higher values correspond to less mobility (Dahl and DeLeire 2008).

**Table 1:** Summary statistics: Mobility and slavery

	A. Full Sample				B. Slave States			
	Mean	Min.	Max.	SD	Mean	Min.	Max.	SD
% Slaves, 1860	15.19	0	88.99	20.92	27.92	0	88.99	21.35
Absolute Upward Mobility (*p* = 25)	42.47	33.10	59.50	4.79	40.24	33.10	51.90	3.64
P(Child in Q5 | Parent in Q1)	8.34	2.20	23.30	3.47	6.89	2.20	18.00	2.83
Relative Mobility (rank-rank slope)	0.34	0.16	0.51	0.06	0.37	0.16	0.51	0.05
CZ per-Year Exposure Effect (*p* = 25)	0.12	-0.91	1.99	0.52	-0.11	-0.91	1.20	0.41

*Notes:* This table reports descriptive statistics for the share of the population enumerated as slaves in the 1860 census and mobility outcomes for the 499 CZs used in the main analysis (panel A) and the 269 CZs located in one of the 15 slave states that existed at the eve of the Civil War (panel B). See the main text for a description of each individual mobility measure.

An additional source of mobility data is Chetty and Hendren (2016a, 2016b), who exploited the movement of families across CZs and the fact that the outcomes for children who move converge with the outcomes for children of permanent residents in the target destination in proportion to the fraction of their childhood spent there. Thus, if a child moves to a CZ where children of permanent residents at similar income levels earn more (less) in adulthood, their expected adult earnings increase (decrease) linearly with the number of years spent there as a child. By exploiting the variation in the age at which children born to parents at the 25th income percentile (*p* = 25) move between CZs, Chetty and Hendren (2016a, 2016b) identified a per-year exposure effect for each CZ that they interpreted as the causal place-level effect on children’s income in adulthood.^6^ As shown in Table 1, panel A, the average percentage change in adult income (at age 26) from spending an additional year as a child in a particular CZ in the sample ranges from an annual loss of 0.91 % to a gain of 1.99 % relative to the national mean, which constitutes a considerable variation as these per-year effects accumulate over a childhood. As shown in panel B of Table 1, on average, the CZs located in the 15 slave states at the eve of the Civil War exhibit substantially worse place-level effects, which is also evident when comparing differences in observed mobility outcomes using the three complementary measures in the rows above.

**Fig. 2:**
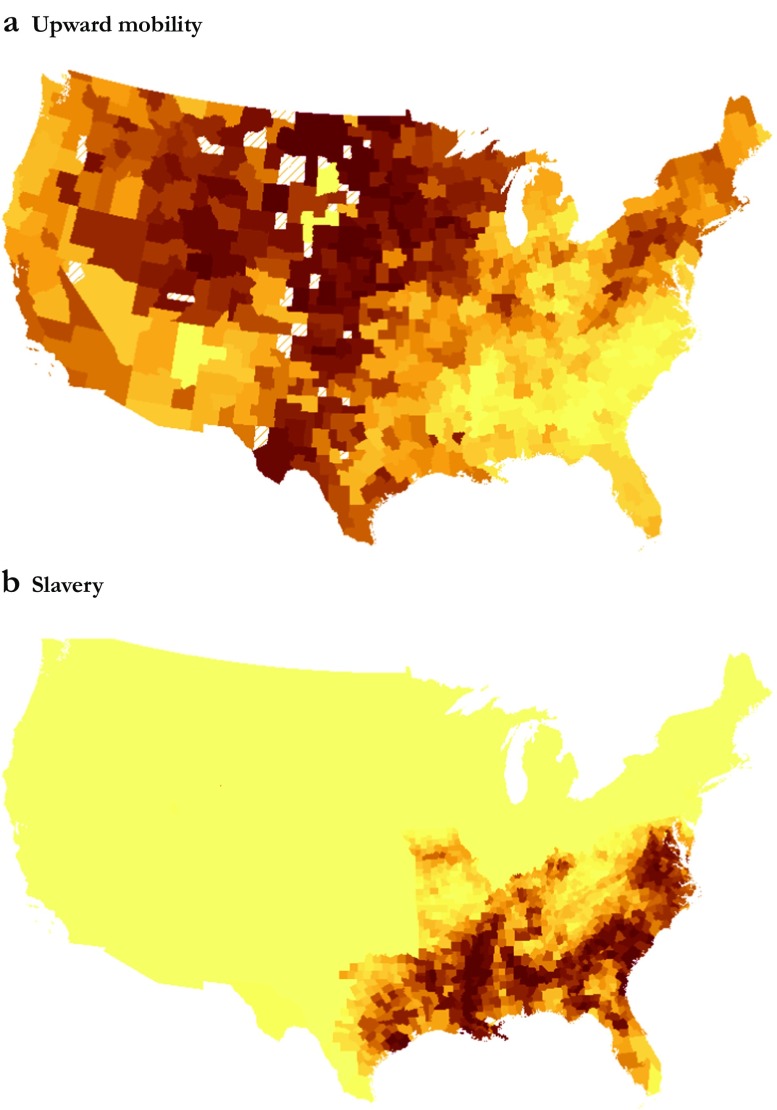
Geography of mobility and slavery in the United States. These maps show differences in absolute upward mobility across CZs measured as the mean income rank at age 30 for children born in the early 1980s (1980–1982) to parents at the 25th percentile of the national income distribution (panel a) and the county-level share of the population that were enumerated as slaves in the 1860 census (panel b). Each map divides the corresponding variable into ventiles, with darker shades denoting higher levels of upward mobility (mobility estimates are unavailable for hatched CZs) and a higher share of slaves, respectively. County (CZ) boundaries are based on maps from IPUMS NHGIS (Manson et al. 2017)

A striking feature of the data is the substantial variation in mobility rates across CZs and the close correspondence between the level of upward mobility and the prevalence of slavery. This is evident from Fig. 2, which maps differences in absolute upward mobility across CZs and the county-level share of the population enumerated as slaves in the 1860 census: darker shades correspond to higher levels of mobility and slave shares, respectively.

**Fig. 3:**
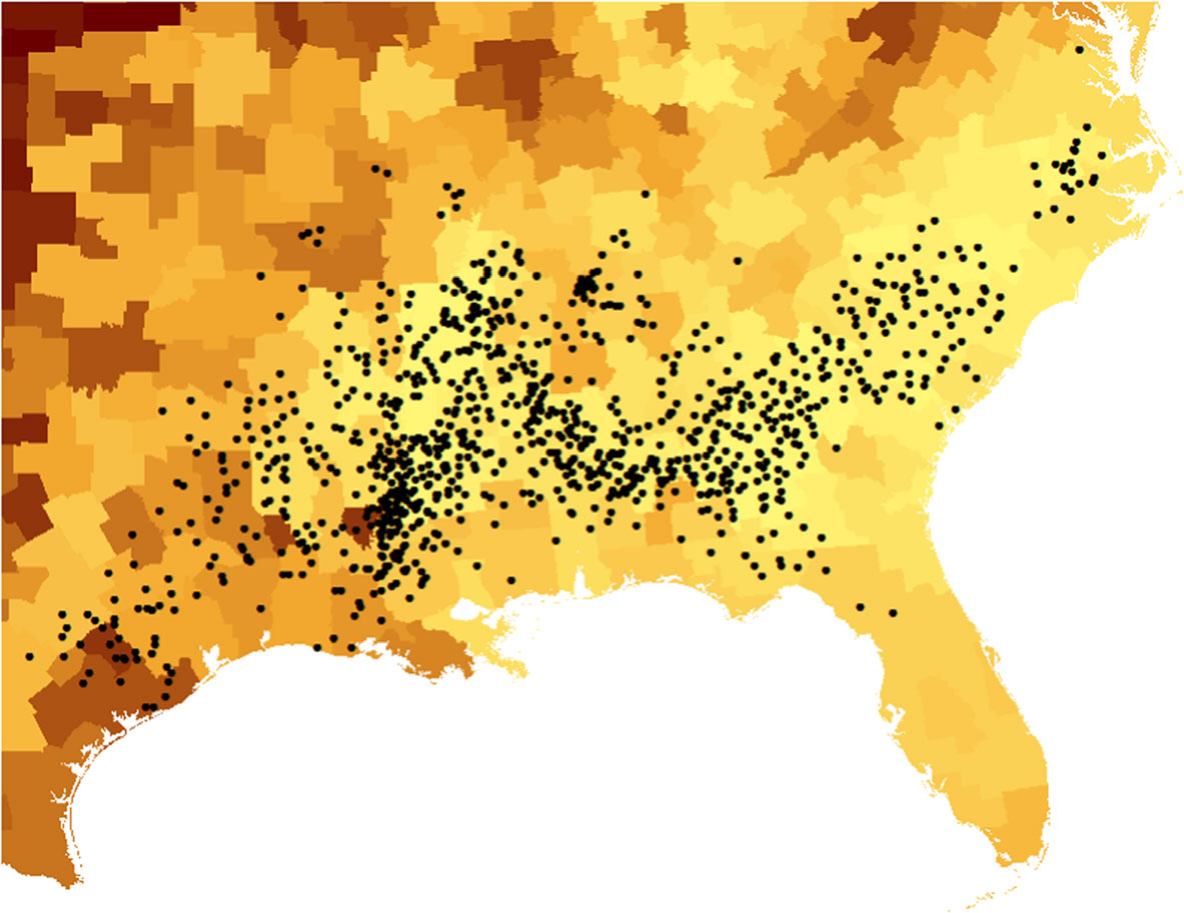
Cotton cultivation and mobility in the American South. This map shows differences in absolute upward mobility across CZs measured as the mean income rank at age 30 for children born in the early 1980s (1980–1982) to parents at the 25th percentile of the national income distribution and cotton output in 1860. Each dot corresponds to approximately 5,000 (400-pound) bales of ginned cotton produced and the underlying mobility data is divided into ventiles, with darker shades corresponding to higher rates of upward mobility. Data on cotton production is drawn from the 1860 Census of Agriculture obtained through IPUMS NHGIS (Manson et al. 2017)

Another indirect link between slavery and upward mobility is depicted in Fig. 3, showing how present-day mobility patterns in the South closely mirror the Cotton Belt that today contains the least upwardly mobile areas in the country. To more systematically examine the potential link between slavery and mobility, I match county-level information on the number of individuals enumerated as slaves in the 1860 census obtained from Haines (2005) to the corresponding CZ using crosswalks available from the U.S. Department of Agriculture’s Economic Research Service, which I then use to calculate the share of the population that were slaves in 1860 in each CZ. Each individual county is matched to a present-day CZ, but because the mobility data are unavailable for a small number of CZs, the main analysis focuses on the 499 CZs for which I observe all four main mobility measures and at least one constituent county exists in the 1860 census.

## Empirical Results

### OLS Estimates

As a starting point to examine the link between slavery and mobility, I estimate ordinary least squares (OLS) regressions on the following form:

1 $$ {M}_c^i=\upalpha +\updelta {S}_c^{1860}+{\lambda}_s+{\mathbf{X}}_c\boldsymbol{\uptheta} +{\upvarepsilon}_c, $$

where ${M}_c^i$ is a mobility measure *i* for CZ *c*, ${S}_c^{1860}$ is the percentage of the population enumerated as slaves in 1860, λ_*s*_ is a full set of state fixed effects, **X**_*c*_ is a vector of CZ–level covariates, and ε_*c*_ is an error term. For the statistical inference, I cluster standard errors at the state level to account for spatial correlation across CZs.

Table 2 presents OLS estimates of Eq. (1), showing that CZs with a higher prevalence of slavery on average exhibit lower upward mobility today.^7^ Each cell corresponds to an individual regression, with standardized coefficients reported in brackets to ease interpretation. As shown in column 1, a 1 standard deviation (SD) increase in the share of the CZ population that were slaves in 1860 is associated with a reduction in absolute upward mobility of 0.589 SDs today—a sizable effect that is also highly statistically significant. Put differently, low-income children growing up in a CZ with a 30 percentage point higher share of the population enumerated as slaves in 1860, which roughly corresponds to the distance between the 50th and 75th percentile of the cross–CZ distribution of slave shares, are predicted to see an average 4.05 (–0.135 × 30) percentile reduction in adult income. As shown in the subsequent two rows of Table 2, the magnitudes remain similar also when examining the chance that a child born in the bottom income quintile ends up in the top quintile, as well as when using the more conventional rank-rank slope measure of relative mobility. While estimated magnitudes are reduced when including state fixed effects in column 2 of Table 2, a large and statistically significant relationship exists between the historical prevalence of slavery and mobility also *within* states, suggesting that these correlations do not exclusively reflect cross-state variation in mobility outcomes.

**Table 2:** Slavery and intergenerational mobility: OLS estimates

	(1)	(2)	(3)	(4)	(5)
Absolute Upward Mobility (*p* = 25)	–0.135**	–0.069**	–0.053**	–0.055**	–0.066**
	(0.020)	(0.009)	(0.012)	(0.010)	(0.011)
	[–0.589]	[–0.301]	[–0.233]	[–0.239]	[–0.385]
P(Child in Q5 | Parent in Q1)	–0.093**	–0.053**	–0.041**	–0.042**	–0.053**
	(0.014)	(0.008)	(0.011)	(0.011)	(0.010)
	[–0.563]	[–0.322]	[–0.245]	[–0.252]	[–0.398]
Relative Mobility (rank-rank slope)	0.002**	0.001**	0.001**	0.001**	0.001**
	(0.0002)	(0.0002)	(0.0002)	(0.0002)	(0.0002)
	[0.592]	[0.451]	[0.411]	[0.397]	[0.438]
CZ per-Year Exposure Effect (*p* = 25)	–0.015**	–0.009**	–0.007**	–0.007**	–0.009**
	(0.002)	(0.002)	(0.002)	(0.001)	(0.002)
	[–0.601]	[–0.346]	[–0.294]	[–0.299]	[–0.452]
State Fixed Effects?	No	Yes	Yes	Yes	Yes
Historical Controls?	No	No	Yes	Yes	Yes
Modern Controls?	No	No	No	Yes	Yes
Sample States	All	All	All	All	Slave states
Number of Observations (CZs)	499	499	499	499	269

*Notes:* This table reports OLS estimates of δ from Eq. (1) where the main right-hand-side variable is the percentage of the population in each CZ that were enumerated as slaves in 1860, and the outcome is one of four measures of absolute or relative mobility listed in the leftmost column of the table and described in more detail in the main text. Column 3 includes CZ–level controls for access to rail/water transportation; the cash value of farms; the share of improved farmland; manufacturing output per capita; population; and the share of the population that are free blacks, employed in manufacturing, and living in urban areas, respectively, based on the 1860 census. Column 4 adds CZ–level controls for average household income and labor force participation rates in 2000, income growth between 2000 and 2010, and whether a CZ intersects a metropolitan area. In column 5, the sample is restricted to CZs that are located in one of the 15 slave states that existed at the eve of the Civil War. Each cell corresponds to an individual regression with standardized coefficients reported in brackets, and robust standard errors clustered at the state level reported in parentheses.

**p < .01

Although these estimates show that CZs with more prevalent slavery exhibit substantially lower levels of upward mobility today, they do not reveal whether this relationship arises due to a causal effect of place or whether it reflects a sorting of families with worse potential mobility outcomes into these areas. Importantly, OLS estimates of the association between slavery and the causal place effect of each CZ reported in the bottom row of Table 2 suggest that a 1 SD increase in the share of slaves in 1860 is associated with a 0.601 SD decrease in the place effect on adult income for low-income children. Put differently, the estimate reported in column 1 suggests that for a child growing up in a CZ with a 30 percentage point higher slave share in 1860, each additional year of childhood spent there leads to an average decrease of 0.45 % (–0.015 × 30) in adult income relative to the national mean; over a childhood of 20 years, this corresponds to a relative decrease of 9.00 % (–0.45 × 20). Overall, this finding suggests that the observed differences in upward mobility largely accrue from causal place effects rather than sorting.

An empirical concern is that the spatial distribution of slavery in 1860 may be correlated with a variety of omitted factors, which in turn may influence differences in mobility.^8^ Therefore, column 3 of Table 2 controls for a wide range of historical development outcomes capturing differences in agricultural development, manufacturing activity, population, transportation, and urbanization.^9^ A similar argument can be made regarding contemporary confounding factors that may reflect a differential development in areas with higher slave shares that is unrelated to slavery itself. To that end, column 4 also controls for a range of modern outcomes, including household income, income growth, labor force participation, and whether a CZ is part of an urban area, although several of these regressors are likely to constitute “bad controls” (Angrist and Pischke 2008).^10^ Last, to shed light on whether the relationship is fully driven by differences between the South relative to other parts of the country, column 5 restricts the analysis to CZs located in one of the 15 slave states, thus identifying the relationship from the intensive rather than extensive margin of slavery. Although these additional controls and sample restrictions lead to moderate declines in estimated magnitudes, a substantial and statistically significant link between a legacy of slavery and lower upward mobility persists, which suggests that this relationship is not mainly reflecting any of these observable factors.^11^

A remaining threat to the validity of these OLS estimates, however, is that the prevalence of slavery may be correlated with unobservable historical or contemporary factors that in turn shape differences in mobility. As a first step to assess this empirical threat, I use the method developed by Altonji et al. (2005) and applied by Nunn and Wantchekon (2011) to assess how large the selection on unobservable characteristics has to be relative to the selection on observable factors in order to explain away the estimated link between slavery and mobility. In practice, it entails estimating two different models: one with a restricted set of regressors, and one with an extensive set of controls. By denoting the estimates in the restricted and full model δ^*R*^ and δ^*F*^, respectively, the ratio δ^*F*^/(δ^*R*^ − δ^*F*^) yields how large the selection on unobservable characteristics has to be relative to the selection on the observable factors included in the full model to render the estimates economically insignificant.

Table 2 presents OLS estimates of the link between absolute upward mobility and the prevalence of slavery that correspond to a bare-bones specification with state fixed effects but without any controls (column 2) and when adding the full set of historical and modern controls, respectively (column 4). Calculating the Altonji et al. (2005) ratio based on these estimates suggests that the selection on unobservable factors needs to be almost four times as large as the selection on observable factors to explain away the results.^12^ Although this exercise thus suggests that unobservable factors must be very influential to explain away the link between slavery and present-day mobility, I next proceed to develop an IV strategy to further reduce concerns that unobservable confounding factors are driving the results.

### IV Estimates

To identify whether the link between slavery and mobility is causal, I exploit differences in the suitability for cultivating cotton as an instrument for the intensity of slavery in 1860 based on data from the FAO’s Global Agro-Ecological Zone (FAO-GAEZ) database taken from Hornbeck and Naidu (2014).^13^ Cotton was the main cash crop cultivated in the South that relied heavily on slave labor, and differences in the suitability for cotton cultivation can therefore be expected to constitute an important determinant of the prevalence of slavery (Acharya et al. 2016) but presumably do not directly affect present-day differences in upward mobility.

**Fig. 4:**
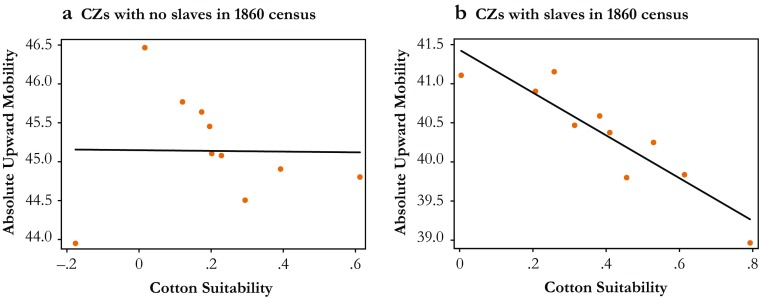
Cotton suitability and upward mobility. These figures show binned scatterplots of the residualized CZ–level link between cotton suitability and absolute upward mobility across CZs with no (0) slaves reported in the 1860 census (panel a) and across CZs with at least one slave reported in the census (panel b). Each group of CZs is collapsed into 10 bins based on the residualized average suitability for cultivating cotton after absorbing state fixed effects, and for each bin, the mean level of residual absolute upward mobility is depicted. The sample means are added back to the residuals of each variable prior to binning and plotting. Also shown are fitted OLS regressions based on the underlying (ungrouped) CZ–level data

A tight link between suitability for cultivating cotton and the use of slave labor is indeed reflected in a strong first-stage relationship between the prevalence of slavery in 1860 and differences in suitability across CZs, as shown in Table 3, panel B.^14^ Although this finding highlights the relevance of the instrument, the exclusion restriction may still be violated if cotton suitability affects mobility through other channels than slavery. To assess the validity of the instrument, I examine whether cotton suitability is uniquely associated with negative mobility outcomes in areas where slavery was widespread. Figure 4 presents binned scatterplots of within-state differences in the mean suitability for cultivating cotton and absolute upward mobility across CZs with no slaves in 1860 according to the census and CZs where at least one slave was recorded. I find no apparent relationship between cotton suitability and upward mobility in CZs with no slavery but a sharply negative relationship in areas where slavery was prevalent. Together, this reduced-form evidence thus suggests that cotton suitability is uniquely associated with lower mobility in areas where slavery was widespread, which indirectly supports the exclusion restriction.

**Table 3:** Slavery and intergenerational mobility: 2SLS estimates

	(1)	(2)	(3)	(4)
A. Outcome: Absolute/Relative Mobility (second stage)				
Absolute upward mobility (*p* = 25)	–0.122*	–0.121^†^	–0.112^†^	–0.106**
	(0.048)	(0.065)	(0.060)	(0.030)
	[–0.534]	[–0.528]	[–0.489]	[–0.622]
P(Child in Q5 | Parent in Q1)	–0.097*	–0.106*	–0.091*	–0.066**
	(0.041)	(0.051)	(0.047)	(0.022)
	[–0.583]	[–0.639]	[–0.550]	[–0.501]
Relative mobility (rank-rank slope)	0.003**	0.004**	0.004**	0.004**
	(0.001)	(0.001)	(0.001)	(0.001)
	[1.197]	[1.431]	[1.371]	[1.522]
CZ per-year exposure effect (*p* = 25)	–0.013*	–0.012^†^	–0.012^†^	–0.012*
	(0.006)	(0.006)	(0.006)	(0.006)
	[–0.518]	[–0.501]	[–0.469]	[–0.614]
B. Outcome: % Slaves, 1860 (first stage)				
Cotton suitability	15.908**	11.703**	11.767**	17.352**
	(3.879)	(3.617)	(3.512)	(4.016)
	[0.267]	[0.197]	[0.198]	[0.281]
State Fixed Effects?	Yes	Yes	Yes	Yes
Historical Controls?	No	Yes	Yes	Yes
Modern Controls?	No	No	Yes	Yes
Kleibergen-Paap *F* Statistic	16.82	10.47	11.23	18.67
Sample States	All	All	All	Slave states
Number of Observations (CZs)	499	499	499	269

*Notes:* Panel A reports two-stage least squares (2SLS) estimates of δ from Eq. (1), where the percentage of the population in each CZ that were enumerated as slaves in 1860 is instrumented with cotton suitability based on data from the FAO-GAEZ, and the outcome is one of four measures of absolute or relative mobility listed in the leftmost column of the table and described in more detail in the main text. Panel B presents the corresponding first stage estimates (across the CZs in the full and restricted sample, the mean cotton suitability is 0.31 (SD = 0.35) and 0.40 (SD = 0.35), respectively). See the notes for Table 2 for a description of the additional controls. Each cell corresponds to an individual regression with standardized coefficients reported in brackets, and robust standard errors clustered at the state level reported in parentheses.

^†^*p* < .10; *p < .05; **p < .01

Panel A of Table 3 presents two-stage least squares (2SLS) estimates of Eq. (1), showing that the negative and highly statistically significant link between slavery and mobility exist also when instrumenting for differences in slave shares across CZs. To further mitigate concerns that the exclusion restriction may be violated, I sequentially include state fixed effects and the full set of historical and modern controls in columns 1–3 and limit the sample to the historical slave states in column 4. Thus, for the exclusion restriction to be violated, the instrument must affect mobility through channels other than, for example, contemporary income or historical differences in industrial development.^15^ Across all four mobility outcomes, the link between slavery and mobility persists, thus suggesting that the correlation documented earlier presumably reflects a causal relationship. Moreover, 2SLS estimates reported in columns 3 and 4 of Table 3 are larger in absolute terms compared with the corresponding OLS estimates reported in columns 4 and 5 of Table 2. This finding is consistent with measurement error in the historical slave data, a downward bias in the OLS estimates due to omitted factors, or that the 2SLS estimates reflect a local average treatment effect. These estimates consistently suggest that areas with more prevalent slavery exhibit substantially lower chances for upward mobility and that these differences arise because of negative causal place effects, raising a pertinent question: *why* do these places produce worse outcomes for low-income children some 150 years after slavery was formally abolished?

### Assessing Potential Channels of Persistence

A good starting point to disentangle the persistent effects of slavery on mobility patterns is to ask whether any of the factors that Chetty et al. (2014a) identified as the most important correlates of mobility across CZs (family structure, income inequality, school quality, segregation, and social capital) can account for the fact that areas with higher slave shares by the outbreak of the Civil War exhibit substantially lower upward mobility today. If any of these factors are relevant to understand this link, we would expect that they vary systematically with the historical distribution of slavery *and* that conditioning on such a factor would reduce the coefficient of the historical slave share.

Panel A of Table 4 presents OLS estimates of Eq. (1) where the outcome is the causal place effect of each CZ on adult income for low-income children, while conditioning on proxies of each potentially mediating factor, respectively.^16^ To ease interpretation, standardized coefficients are again reported in brackets. Similar regressions in which each factor listed in the column heads is used as an outcome are reported in panel B to examine the extent to which they correlate with the prevalence of slavery. To reduce concerns that the variation in the potentially mediating factors is mainly driven by unique features of places in the South, I focus here on the subsample of CZs that are located in the 15 slave states and include the full set of historical and modern controls, as well as state fixed effects.^17^

**Table 4:** Explanation of the link between slavery and mobility

	Segregation		Inequality		Social Capital	K–12 School System				Family Structure		
	Income	Race	Gini	Top 1 %	R & G Index	High School Dropout Rate	Test Scores	Student-Teacher Ratio	Expenditure	Divorced	Married	Single Mothers
	(1)	(2)	(3)	(4)	(5)	(6)	(7)	(8)	(9)	(10)	(11)	(12)
A. Conditional Effect of Slavery on Mobility (outcome: CZ per-year exposure effect (*p* = 25))												
% slaves, 1860	–0.007**	–0.008**	–0.008**	–0.009**	–0.008**	–0.008**	–0.006**	–0.008**	–0.009**	–0.009**	–0.004**	–0.001
	(0.001)	(0.001)	(0.001)	(0.002)	(0.002)	(0.002)	(0.001)	(0.002)	(0.002)	(0.002)	(0.001)	(0.001)
	[–0.375]	[–0.438]	[–0.425]	[–0.462]	[–0.436]	[–0.390]	[–0.326]	[–0.422]	[–0.451]	[–0.457]	[–0.233]	[–0.032]
Factor in column head	–0.042**	–0.010**	–0.014**	–0.006	0.060	–0.048*	0.018**	–0.047	–0.001	–0.010	0.036**	–0.045**
	(0.007)	(0.003)	(0.005)	(0.006)	(0.049)	(0.019)	(0.004)	(0.035)	(0.001)	(0.020)	(0.005)	(0.005)
	[–0.330]	[–0.194]	[–0.229]	[–0.065]	[0.117]	[–0.226]	[0.296]	[–0.163]	[–0.108]	[–0.031]	[0.395]	[–0.604]
B. Effect of Slavery on Modern Outcomes (outcome: listed in column heads)												
% slaves, 1860	0.035*	0.027	0.036	–0.030	–0.005	0.020*	–0.134*	0.004	0.026	–0.010*	–0.119**	0.179**
	(0.014)	(0.035)	(0.039)	(0.019)	(0.004)	(0.008)	(0.049)	(0.006)	(0.126)	(0.005)	(0.022)	(0.024)
	[0.235]	[0.072]	[0.116]	[–0.155]	[–0.135]	[0.219]	[–0.424]	[0.063]	[0.009]	[–0.170]	[–0.555]	[0.695]
Mean (SD) of Factor in column head	4.41	14.43	46.33	11.72	–0.77	0.65	–3.02	16.37	538.10	9.97	56.44	23.36
	(3.22)	(7.84)	(6.56)	(4.12)	(0.80)	(1.96)	(6.73)	(1.48)	(65.22)	(1.29)	(4.57)	(5.50)
State Fixed Effects?	Yes	Yes	Yes	Yes	Yes	Yes	Yes	Yes	Yes	Yes	Yes	Yes
Historical Controls?	Yes	Yes	Yes	Yes	Yes	Yes	Yes	Yes	Yes	Yes	Yes	Yes
Modern Controls?	Yes	Yes	Yes	Yes	Yes	Yes	Yes	Yes	Yes	Yes	Yes	Yes
Number of Observations (CZs)	269	269	269	269	269	258	268	244	269	269	269	269

*Notes:* Panel A reports OLS estimates of Eq. (1), where the main right-hand-side variable is the percentage of the population enumerated as slaves in 1860, and the outcome is the causal place effect of each CZ, while conditioning on each factor listed in the column heads. Panel B presents similar OLS regressions with each factor listed in the column heads as the outcome, with the mean (SD) of each outcome reported in the bottom of the table. Income and racial segregation is measured as a rank-order index estimated at the census-tract level and a multigroup Theil index based on four groups (black, Hispanic, other, and white), respectively, both based on data from the 2000 census and both scaled by a factor of 100 for presentational purposes. Inequality is measured by the Gini coefficient of income (× 100), and the share of income accruing to the top 1 % based on tax records for the sample used to derive mobility rates. Social capital is measured by the Rupasingha and Goetz (2008) standardized index that combines measures of voter turnout rates, the share of people returning their census forms, and participation in community organizations. K–12 schooling characteristics are: a residual of high school dropout rates (× 100) and mean math and English standardized test scores after regressing them on household income per capita in 2000, the average student-teacher ratio in public schools, and the average expenditure per student (× 100) based on data from the NCES CCD and the George Bush Global Report Card. Family structure is measured by the percentage of the population aged 15 and older who are divorced and married (and not separated), respectively, and the percentage of all households with children that are headed by a single mother based on the 2000 census. See Chetty et al. (2014a) for a further description of these variables and see the notes to Table 2 for a description of the additional controls. Standardized coefficients are reported in brackets, and robust standard errors clustered at the state level are reported in parentheses.

*p < .05; **p < .01

In panel A of Table 4, columns 1–4 show that the estimated link between slavery and mobility remains similar in magnitude when conditioning on segregation by income or race as well as two alternative measures of income inequality (the Gini coefficient and the share of income accruing to the top 1 %) relative to the corresponding OLS estimate without these additional covariates (see Table 2, column 5).^18^ Similarly, controlling for differences in the level of social capital as reflected in the index of Rupasingha and Goetz (2008) does not seem to affect the link between slavery and upward mobility (column 5 of Table 4).^19^

An emphasis on the role of the educational system in shaping mobility outcomes is broadly supported by the results in columns 6–9 of Table 4, which condition on four aspects of the K–12 school system: dropout rates and test scores adjusted for parental income, and the average student-teacher ratio and expenditure per student in public schools.^20^ Yet, although output-based measures of school quality (dropout rates and test scores) are significantly worse in areas with more prevalent slavery (panel B, columns 6 and 7), it does not seem to account for the reduced-form link between slavery and upward mobility (panel A).

A final factor that has been highlighted as a key determinant of mobility patterns is differences in family structure. Columns 10–12 of Table 4 condition on three measures of the stability of family structures: the percentage of the population aged 15 and older that are divorced and married, respectively, as well as the percentage of households with an own child present and headed by single mothers. When conditioning on the share of female single-parent households in column 12, the association between slavery and upward mobility loses its statistical significance, and the coefficient is effectively reduced to 0, which is consistent with a significant part of the lingering effect of slavery working through more fragile family structures in these places. A link between slavery and subsequently more fragile family structures is further reinforced by the estimates presented in panel B of Table 4, which show that a 1 SD increase in slavery is associated with a 0.555 SD decrease in the share married and an even larger (0.695 SD) increase in the share of children living in single-parent households (columns 11 and 12).^21^ Although it is beyond the scope of this study to identify how slavery affected the evolution of family structures over the twentieth century, it is interesting to note that these results are broadly consistent with prior research that has emphasized how the legacy of slavery shaped family structures after the Civil War and the historical continuity of family patterns (e.g., Gordon and McLanahan 1991; Miller forthcoming; Morgan et al. 1993; Ruggles 1994).

## Conclusions

A key contribution of this article is to document that the present-day geography of intergenerational mobility in the United States largely reflects the historical distribution of slavery, with substantially less upward mobility in areas with a higher share of slaves by the outbreak of the Civil War. Based on a variety of empirical strategies, the evidence suggests that this relationship is causal. Exploiting differences reported by Chetty and Hendren (2016a, 2016b) in observed mobility rates for children whose families move across CZs to identify the place-based component of upward mobility suggests that this relationship does not arise mainly from sorting of families across CZs; rather it reflects a causal effect of place.

As a starting point to understand why slavery still shapes the geography of opportunity in the United States, I examine whether the five broad factors highlighted by Chetty et al. (2014a) as the most important correlates of mobility can account for the documented link between slavery and upward mobility. More fragile family structures in areas that had more prevalent slavery is seemingly the most important for understanding why these places produce significantly worse mobility outcomes today. Although these results are suggestive, they should be interpreted carefully because of the extremely challenging task of identifying the wide variety of causal transmission mechanisms that may link slavery to present-day differences in mobility. Further work is necessary for understanding how these differences emerged and the extent to which they link the past to the present.

## Footnotes

1 See Aaronson and Mazumder (2008), Long and Ferrie (2013), Clark (2014), and Chetty et al. (2017) for a longer-term view on mobility in the United States; see Solon (1999) and Black and Devereux (2011) for an overview of the extensive body of work on intergenerational mobility.

2 CZs correspond to local labor markets, which are identified as clusters of counties that exhibit strong commuting ties within and weak ties between clusters (see Tolbert and Sizer 1996).

3 Parental incomes are measured in 1996–2000, and child incomes are measured in 2011–2012.

4 See http://www.equality-of-opportunity.org.

5 Because the rank-rank relationship is approximately linear, the outcomes for children born to parents at the 25th percentile correspond to the average income rank in adulthood for children born in the bottom half of the income distribution.

6 As Chetty and Hendren (2016a, 2016b) extensively documented, these estimates remain similar also when, for example, controlling for a range of factors that may have triggered parental moves that may be correlated with potential outcomes for children, exploiting sibling variation in exposure, or using exogenous shocks to migration decisions.

7 Although I present unweighted regressions throughout the article, results remain broadly similar in magnitude and statistical precision when weighting the reported regressions by CZ populations in either 1860 or 2000, or by the number of children in the 1980–1982 birth cohorts (not reported).

8 Although race is not observed for individuals in the data, the link between slavery and mobility exists also when focusing on cross–CZ differences in mobility rates restricted to individuals living in ZIP codes where at least 80 % of the population are non-Hispanic whites. Among the 449 CZs for which these data are available, the OLS estimate (standard error) from regressing absolute upward mobility in “white” ZIP codes on the percentage of slaves in 1860 is –0.084 (0.026), which is smaller in absolute terms than the estimate in column 1 of Table 2 but still sizable and statistically significant. Furthermore, Chetty et al. (2014a) reported that the unweighted correlation between mobility for predominantly whites and the full sample is 0.91, which suggests that the observed CZ–level differences in upward mobility do not mainly reflect individual-level race differences in mobility.

9 From the 1860 census, I calculate the (ln) population, the share of the population that are free blacks, and the share living in cities with at least 2,500 inhabitants. As proxies for agricultural development, I calculate the (ln) cash value of farms and the share of improved farmland of total (improved and unimproved) farmland. Last, I create a dummy variable indicating whether any county in a CZ had access to water or rail transportation, and calculate the share employed in manufacturing and the (ln) manufacturing output per capita, respectively.

10 To account for differences in economic structure today, I include the average household income and labor force participation in 2000, income growth between 2000 and 2010, and a dummy variable for whether a CZ intersects a metropolitan area from Chetty et al. (2014a). As suggested by an anonymous referee, the long-term effects of slavery may also reflect differences in geographical mobility across CZs. Controlling for contemporary migration in/outflows in each CZ, however, does little to affect the link between slavery and mobility (not reported).

11 Sokoloff and Engerman (2000) argued that (land) inequality is intrinsically linked to slavery and to subsequent economic development. To evaluate whether historical inequality accounts for the effect of slavery and whether it directly shapes present-day patterns of mobility, I construct a Gini coefficient of the farm size distribution using a procedure similar to that used by Nunn (2008b) with data from the 1860 census. In a subsample of CZs (*n* = 458) for which I observe land inequality, the link between slavery and lower upward mobility remains highly statistically significant and similar in magnitude across all four outcomes when adding the Gini coefficient to similar specifications as in column 4 of Table 2, but land inequality itself is seemingly unrelated to present-day patterns of mobility (not reported).

12 Based on the estimates in Table 2, columns 2 and 4, the ratio is calculated as −0.055 / ((−0.069) − (−0.055)) = 3.93. Similar exercises can be carried out for the other outcomes reported in Table 2. Consistently, the high Altonji et al. (2005) ratios (3.50–11.00) suggest that selection on unobservable factors needs to be very influential relative to the observable factors included to explain away the estimated effect.

13 Cotton suitability reflects the mean suitability for cultivating cotton based on county-level estimates on climate, growing conditions, and soil types as defined in the FAO-GAEZ database (version 3.0). Maximum potential yields are based on climate averages between 1961 and 1990 and assuming rain-fed conditions and intermediate input levels.

14 As indicated in the bottom of Table 3, the Kleibergen-Paap *F* statistic from the first stage is sufficiently large to reduce weak instrument concerns in all four specifications.

15 A particular concern is that differences in the suitability for cultivating cotton may be correlated with other geographic features that in turn may have affected long-term development patterns. However, although controlling for other geographic characteristics (such as altitude and ruggedness) from the USGS National Elevation Dataset in the first stage somewhat weakens the power of the instrument, the second stage estimates are of a similar magnitude and remain highly statistically significant (not reported).

16 For brevity, I focus here on the causal place effect as an outcome, although using the alternative measures of absolute and relative mobility produces very similar results (not reported). Additional CZ characteristics are drawn from Chetty et al. (2014a) and are described in more detail in the notes for Table 4.

17 Results are, however, similar in the full sample of CZs (not reported).

18 Although such comparisons should be made with care, it is interesting to note that slavery has a larger effect on contemporary mobility rates (in SD terms) relative to influential factors such as income inequality or segregation. Similarly, alternative measures of (income) segregation derived by Chetty et al. (2014a)—such as the extent to which the bottom and top income quartiles are isolated from families at higher and lower income levels, respectively—do not affect the estimated link between slavery and mobility (not reported).

19 Similarly, controlling for alternative measures of social capital used by Chetty et al. (2014a), such as the fraction of religious individuals or violent crime rates, does not alter the link between slavery and mobility (not reported).

20 A similar analysis of the potential role for higher education reflected in the number of Title IV institutions per capita, the average in-state fees/tuition for first-time undergraduates, or the income-adjusted college graduation rates in a CZ leaves the link between slavery and mobility virtually unaffected, suggesting that this relationship does not reflect differences in the provision of higher education (not reported). The K–12 characteristics are not available for all CZs, which slightly reduces the number of observations in columns 6–8 of Table 4.

21 An alternative explanation for the higher share of single-parent households in areas with more prevalent slavery may be that this partly reflects a historical concentration of African Americans in these CZs. Indeed, the raw correlation between the share of single-parent households and the share of black residents is 0.93, and controlling for black shares renders the link between slavery and mobility statistically insignificant. However, the link between the CZ-level share of black residents and upward mobility turns *positive* albeit not statistically significant when I simultaneously control for the share of single-parent households, whereas the latter remains an economically and statistically significant correlate of lower upward mobility. Chetty et al. (2014a) similarly documented this relationship and argued that it provides suggestive evidence that the link between race and mobility is likely to work through indirect channels (e.g., differences in family structure) rather than through a direct effect of race on mobility. This possibility is consistent with the evidence described earlier showing that mobility rates are also lower in predominately “white” ZIP codes in areas with more prevalent slavery and the results reported throughout this article showing that most of these effects seemingly arise at the community level.

## References

[CR1] Aaronson D, Mazumder B (2008). Intergenerational economic mobility in the United States, 1940 to 2000. Journal of Human Resources.

[CR2] Acharya A, Blackwell M, Sen M (2016). The political legacy of American slavery. Journal of Politics.

[CR3] Altonji JG, Elder TE, Taber CR (2005). Selection on observed and unobserved variables: Assessing the effectiveness of Catholic schools. Journal of Political Economy.

[CR4] Angrist JD, Pischke J-S (2008). Mostly harmless econometrics: An empiricist’s companion.

[CR5] Bertocchi G, Dimico A (2014). Slavery, education, and inequality. European Economic Review.

[CR6] Black SE, Devereux PJ (2011). Recent developments in intergenerational mobility. Handbook of Labor Economics.

[CR7] Chetty R, Grusky D, Hell M, Hendren N, Manduca R, Narang J (2017). The fading American dream: Trends in absolute income mobility since 1940. Science.

[CR8] Chetty R, Hendren N (2016). *The impacts of neighborhoods on intergenerational mobility I: Childhood exposure effects* (NBER Working Paper No. 23001).

[CR9] Chetty R, Hendren N (2016). *The impacts of neighborhoods on intergenerational mobility II: County-level estimates* (NBER Working Paper No. 23002).

[CR10] Chetty R, Hendren N, Kline P, Saez E (2014). Where is the land of opportunity? The geography of intergenerational mobility in the United States. Quarterly Journal of Economics.

[CR11] Chetty R, Hendren N, Kline P, Saez E, Turner N (2014). Is the United States still a land of opportunity? Recent trends in intergenerational mobility. American Economic Review: Papers and Proceedings.

[CR12] Clark G (2014). The son also rises: Surnames and the history of social mobility.

[CR13] Collins WJ, Wanamaker MH (2015). *Up from slavery? Intergenerational mobility in the shadow of Jim Crow* (NBER Working Paper No. 23395).

[CR14] Corak M (2013). Income inequality, equality of opportunity, and intergenerational mobility. Journal of Economic Perspectives.

[CR15] Dahl MW, DeLeire T (2008). *The association between children’s earnings and fathers’ lifetime earnings: Estimates using administrative data* (Discussion Paper No. 1342-08).

[CR16] Dell M (2010). The persistent effects of Peru’s mining “mita.”. Econometrica.

[CR17] Gordon L, McLanahan S (1991). Single parenthood in 1900. Journal of Family History.

[CR18] Haines MR (2005). *Historical, demographic, economic, and social data: The United States, 1790–2000* [Data sets from ICPSR 2896].

[CR19] Hornbeck R, Naidu S (2014). When the levee breaks: Black migration and economic development in the American south. American Economic Review.

[CR20] Lagerlöf, N.-P. (2005). *Geography, institutions and growth: The United States as a microcosm*. Unpublished manuscript, Department of Economics, York University, Toronto, Ontario, Canada.

[CR21] Lee C-I, Solon G (2009). Trends in intergenerational income mobility. Review of Economics and Statistics.

[CR22] Long J, Ferrie J (2013). Intergenerational occupational mobility in Great Britain and the United States since 1850. American Economic Review.

[CR23] Manson S, Schroeder J, van Riper D, Ruggles S (2017). *IPUMS National Historical Geographic Information System: Version 12.0* [Data set].

[CR24] Mazumder B (2005). Fortunate sons: New estimates of intergenerational mobility in the United States using social security earnings data. Review of Economics and Statistics.

[CR25] Miller, M. C. (Forthcoming). Destroyed by slavery? Slavery and African American family formation following emancipation. *Demography*. 10.1007/s13524-018-0711-6 30218275

[CR26] Mitchener KJ, McLean IW (2003). The productivity of US states since 1880. Journal of Economic Growth.

[CR27] Morgan SP, McDaniel A, Miller AT, Preston SH (1993). Racial differences in household and family structure at the turn of the century. American Journal of Sociology.

[CR28] Nunn N (2008). The long-term effects of Africa’s slave trades. Quarterly Journal of Economics.

[CR29] Nunn N (2008). Slavery, inequality, and economic development in the Americas.

[CR30] Nunn, N. (2009). The importance of history for economic development. *Annual Review of Economics, 1,* 65–92. 10.1146/annurev.economics.050708.143336

[CR31] Nunn N, Wantchekon L (2011). The slave trade and the origins of mistrust in Africa. American Economic Review.

[CR32] Olivetti C, Paserman MD (2015). In the name of the son (and the daughter): Intergenerational mobility in the United States, 1850–1940. American Economic Review.

[CR33] Ruggles S (1994). The origins of African-American family structure. American Sociological Review.

[CR34] Rupasingha A, Goetz SJ (2008). *US county-level social capital data, 1990–2005* [Data set].

[CR35] Sacerdote B (2005). Slavery and the intergenerational transmission of human capital. Review of Economics and Statistics.

[CR36] Sokoloff KL, Engerman SL (2000). History lessons: Institutions, factors endowments, and paths of development in the New World. Journal of Economic Perspectives.

[CR37] Solon G (1992). Intergenerational income mobility in the United States. American Economic Review.

[CR38] Solon G (1999). Intergenerational mobility in the labor market. Handbook of Labor Economics.

[CR39] Tolbert CM, Sizer M (1996). *U.S. commuting zones and labor market areas: A 1990 update* (ERS Staff Paper No. 9614).

